# Supplementary figures and images for: Modeling cancer genomic data in yeast reveals selection against ATM function during tumorigenesis
Source: PLoS Genet. 2020 Mar 18;16(3):e1008422. doi: 10.1371/journal.pgen.1008422 (PMC7105138; doi:10.1371/journal.pgen.1008422)

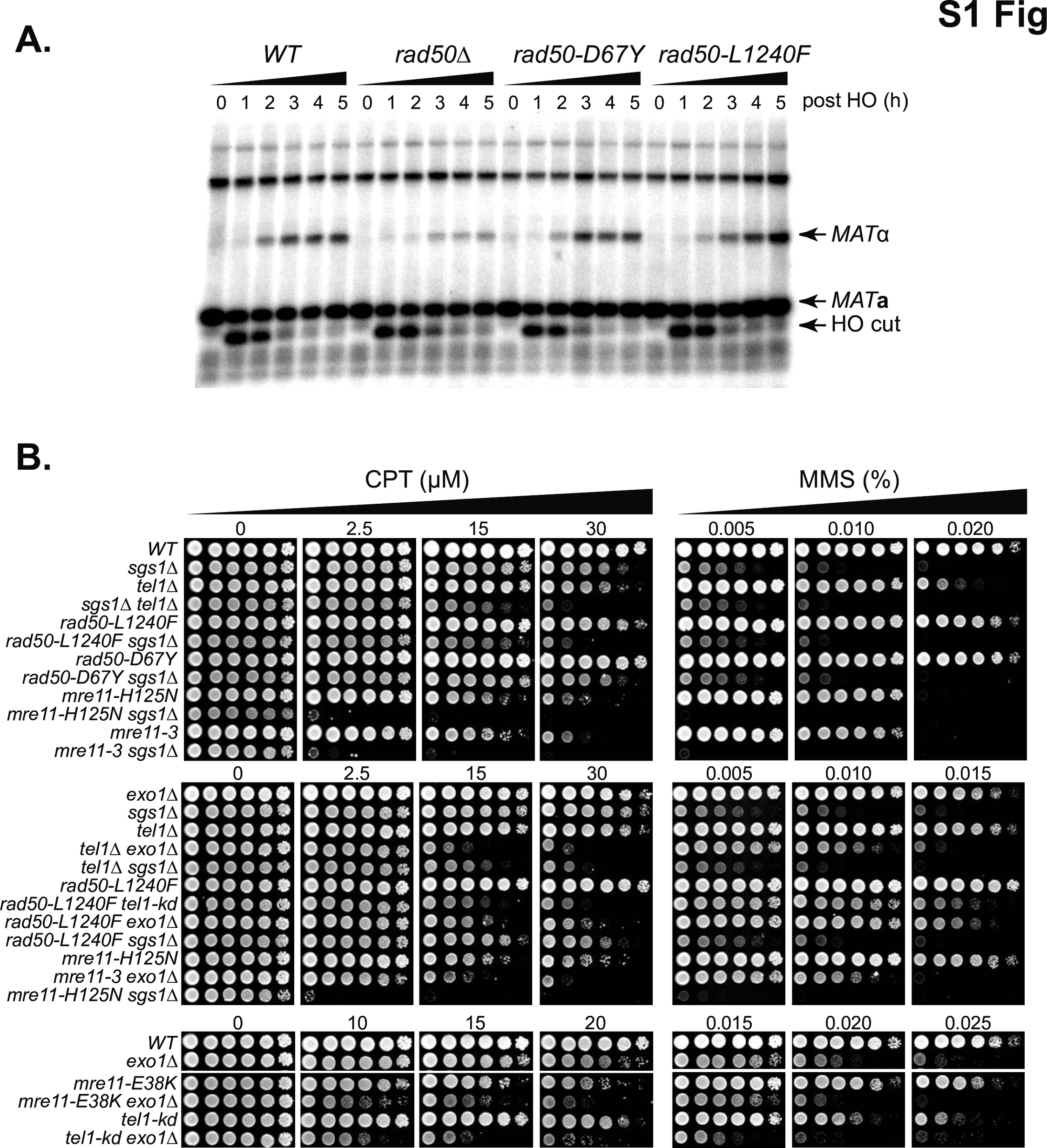

Supplement: S1 Fig — Figure related to Fig 2. (A) Mating type switching assay to measure HO-DSB by homologous recombination. HO-DSB formation was induced for one hour in MATa cells and HO-DSB repair was monitored by southern blot over 4 hours using StyI-digested genomic DNA and a MAT locus specific probe (chromosome III coordinates 201176 to 201580) as previously described [65]. The HO-endonuclease produces a 0.7 kb fragment (HO-cut, indicated by arrow) from the 0.9 kb MATa StyI fragment (indicated by an arrow) and a 1.8 kb StyI-fragment is produced upon homologous recombination repair of the HO-DSB with the MATα donor template (indicated by an arrow). Supporting Methods are given in S1 Text. (B). Clastogen survival of rad50-L1240F, rad50-D67Y and mre11-E38K in sgs1Δ and/or exo1Δ background. For comparison, tel1Δ and mre11 nuclease dead alleles (mre11-H125N or mre11-3) were included. (TIF) [file pgen.1008422.s001.tif]

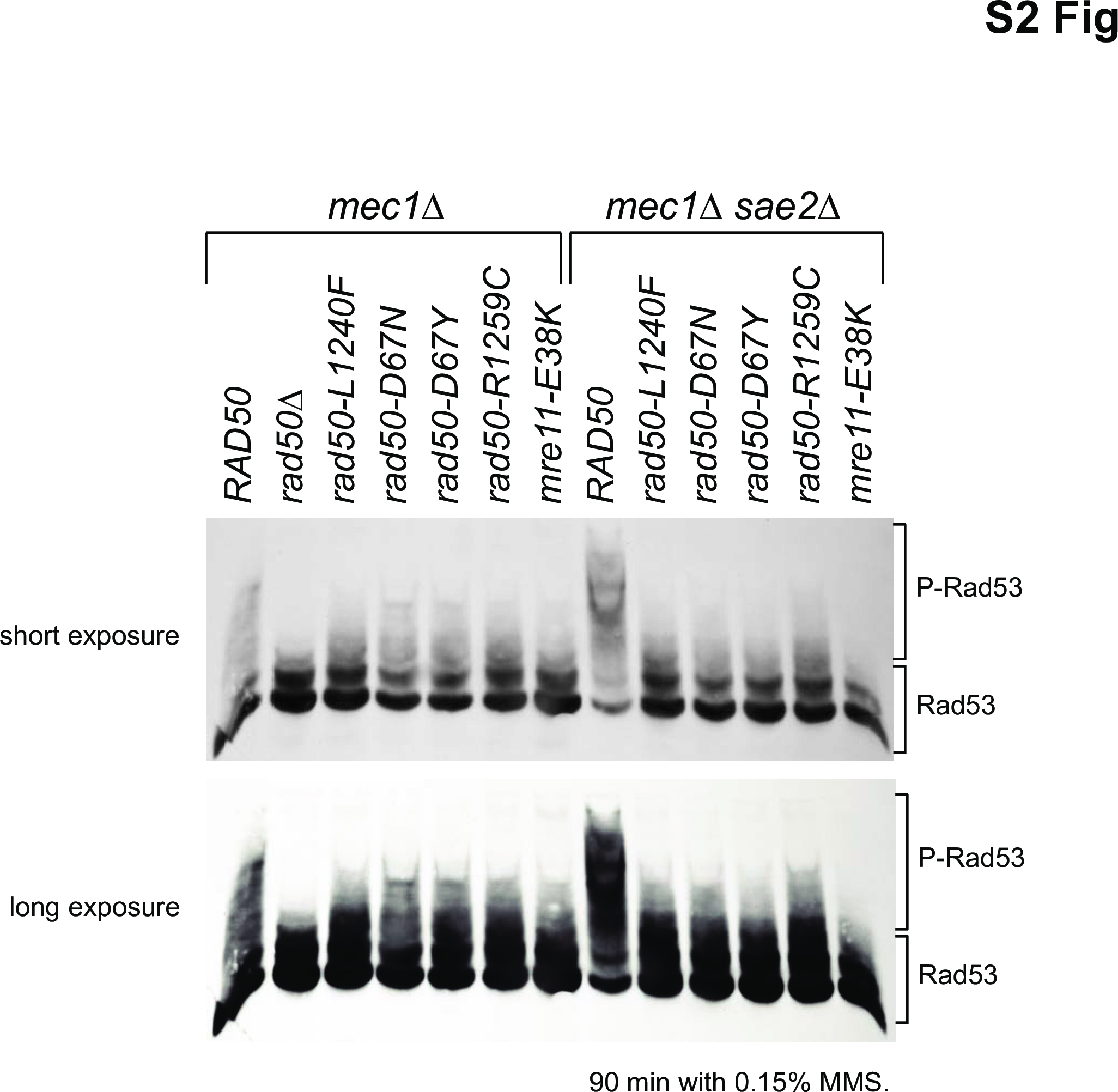

Supplement: S2 Fig — Figure related to Fig 3B. Exponentially growing cells were treated for 90 min with 0.15% MMS and 10 μg TCA-extracts were run on a 8% PAA gel (14x16 cm) containing 20 μM Phos-tag (Fujifilm Wako Pure Chemical Corporation, AAL-107) and 40 μM MnCl2 for 16 hours at 50V at RT. The gel was soaked for 20 min in 10 mM EDTA, transferred to PVDF membrane and probed sequential with FLAG M2 antibody and anti-mouse HRP. A short and long exposure of the same membrane is shown. (TIF) [file pgen.1008422.s002.tif]

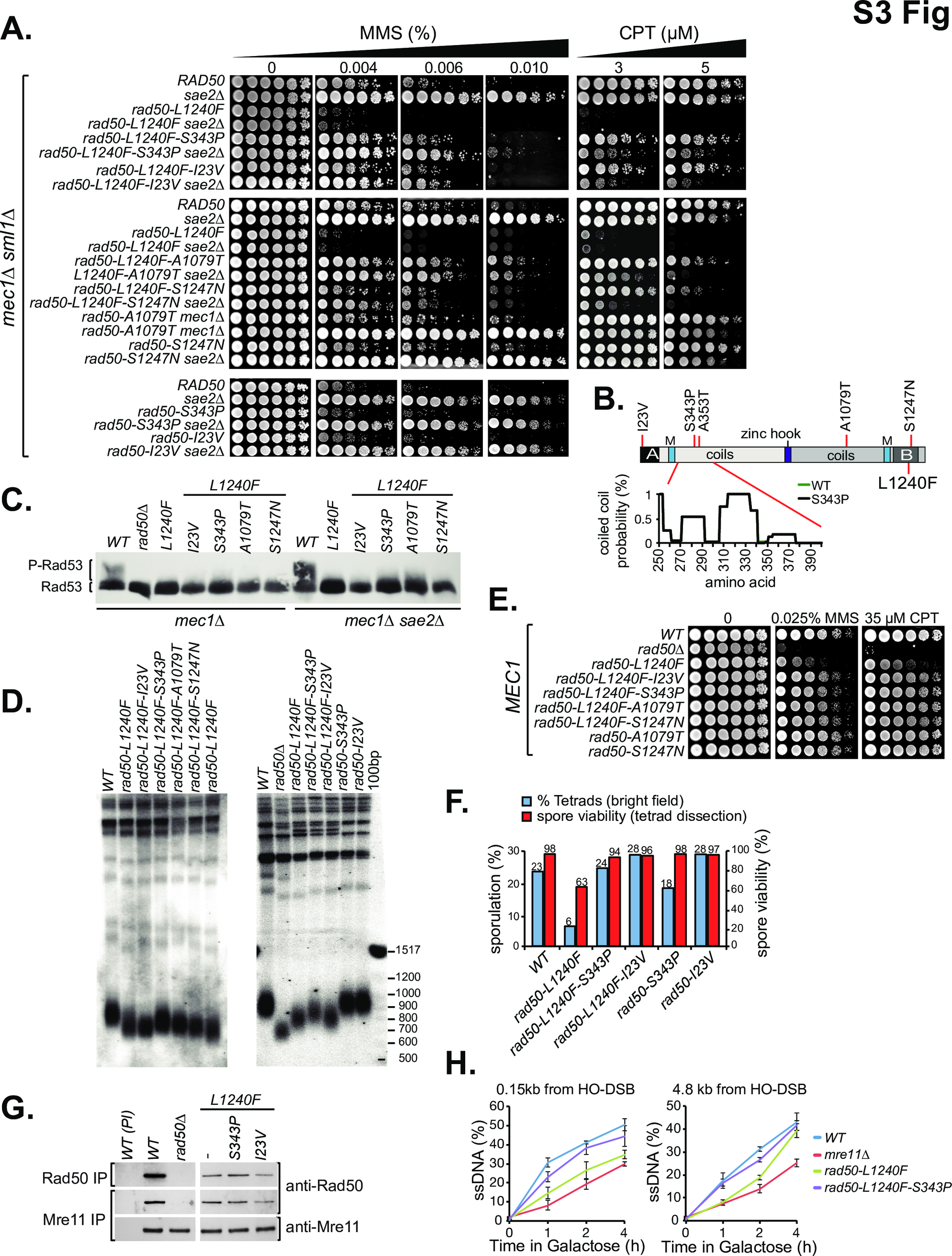

Supplement: S3 Fig — Please see also S2 Text for a more detailed description. (A) MMS- and CPT-survival of rad50-L1240F suppressor mutants S343P, I23V, A1079N and S1247N in mec1Δ and mec1Δ sae2Δ background. (B) Identified rad50-L1240F intragenic suppressors denoted on Rad50 primary structure. (C) Tel1-dependent Rad53 phosphorylation in mec1Δ and mec1Δ sae2Δ cells after 90 min treatment with 0.1% MMS (+) was assessed by western blotting with anti-Flag-Rad53. Migration levels of the non-phosphorylated form (Rad53) and the phosphorylated form (P-Rad53) are indicated. Accordingly to survival in mec1Δ and mec1Δ sae2Δ background, the suppressor subtly mitigated the Tel1-dependent Rad53-phoshorylation defect of rad50-L1240F. (D). Telomere southern blots. Effect of rad50-L1240F intragenic suppressors on telomere lengths. Telomere lengths were assessed of freshly dissected spores after 30 generation of growth with either PstI digested (blot on right) or XhoI-digested (blot on left) genomic. Size makers are only given for one of the blots. (E) MMS- and CPT-survival of rad50-L1240F intragenic suppressors in Mec1-proficient background. (F). rad50-L1240F partial meiotic phenotypes is suppressed to WT levels in rad50-L1240F-S343P and rad50-L1240F-I23V diploids. Sporulation efficiency (left axis) and spore viability (right axis) are plotted. (G) Mre11 complex integrity of rad50-L1240F without and with intragenic suppressors assessed by Rad50 and Mre11 immunoprecipitation and western blotting. (H) Q-PCR based resection assay. The S343P suppressor alleviates rad50-L1240F reduced DSB resection. Error bars denote standard deviation from three experiments. Other suppressors were not assessed. (TIF) [file pgen.1008422.s003.tif]

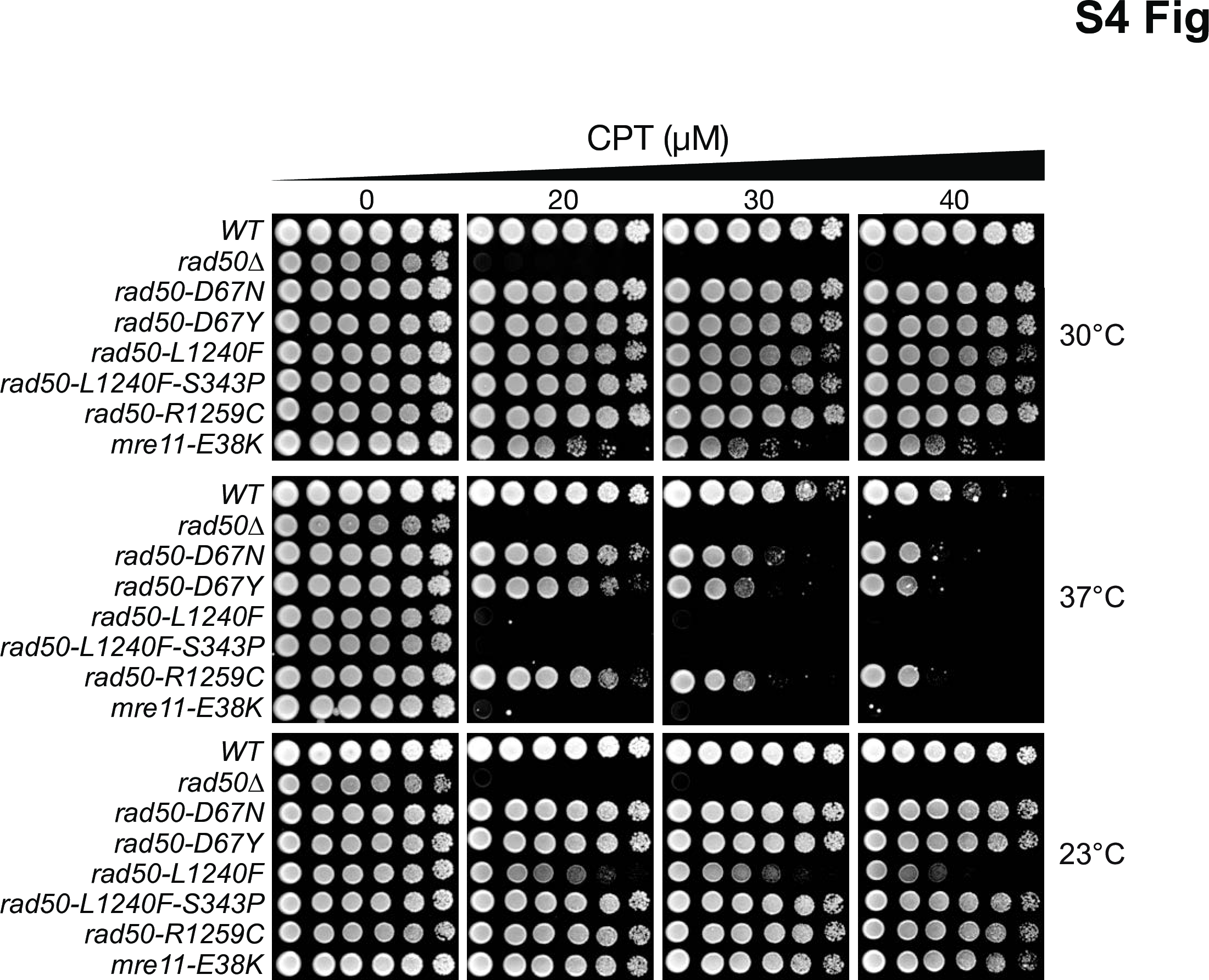

Supplement: S4 Fig — Figure related to Fig 2A. Indicated strains were incubated at 30°C or 37°C for 2 days or at 23°C for 4 days. (TIF) [file pgen.1008422.s004.tif]

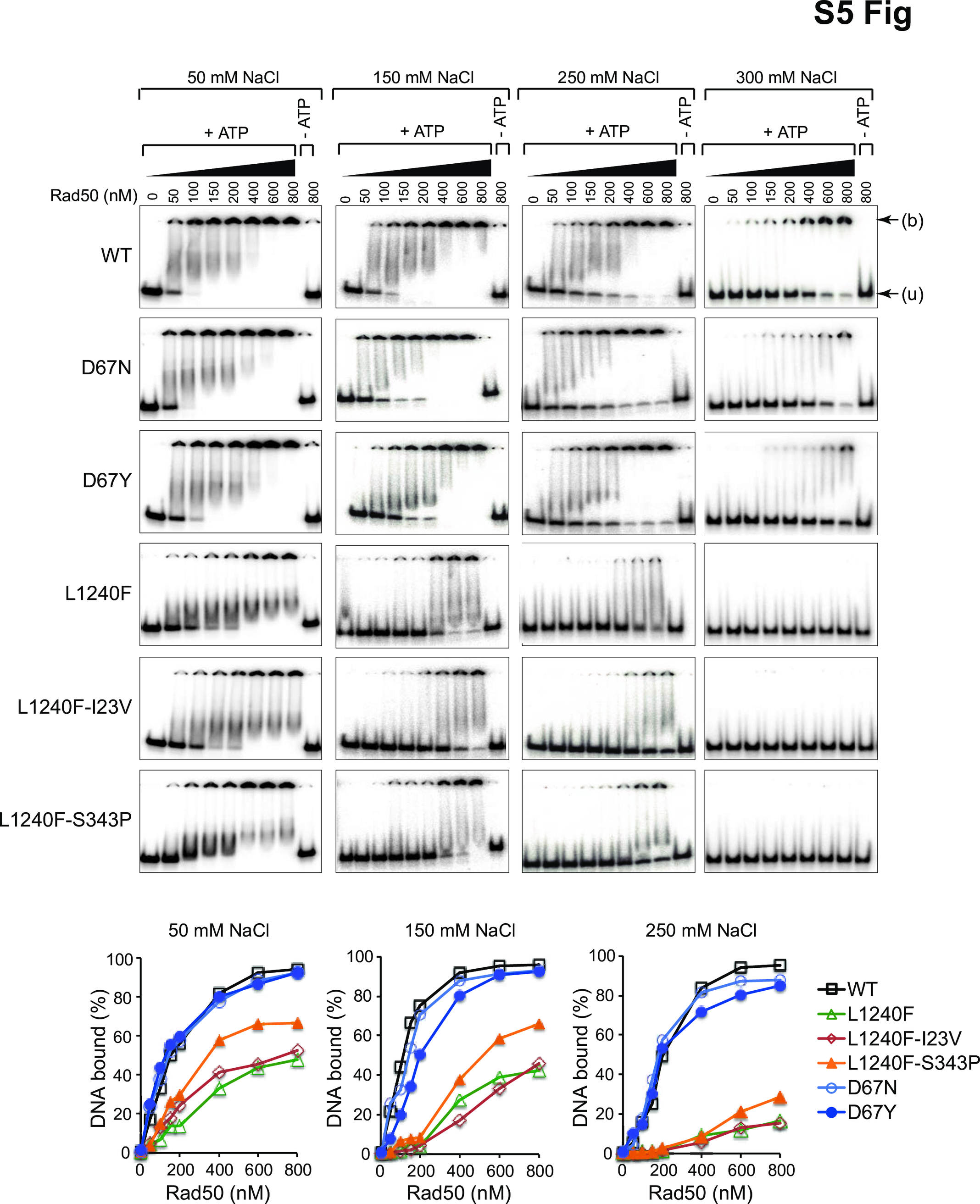

Supplement: S5 Fig — Figure related to Fig 4C. Rad50 dsDNA binding was assessed was assessed at 50 mM, 150 mM, 250 mM and 300 mM NaCl. Increasing concentrations of Rad50 (0–800 nM) were incubated in a binding buffer containing the indicated concentrations of NaCl with 5 nM of a 32P-labeled 83-mer dsDNA oligonucleotide in presence of ATP and MgCl2 or absence of ATP (assessed only for 800 nM Rad50). The migration levels of the unbound (u) and Rad50 bound (b) DNA substrate is denoted. A quantification of the shown EMSA gels is given (on bottom). (TIF) [file pgen.1008422.s005.tif]

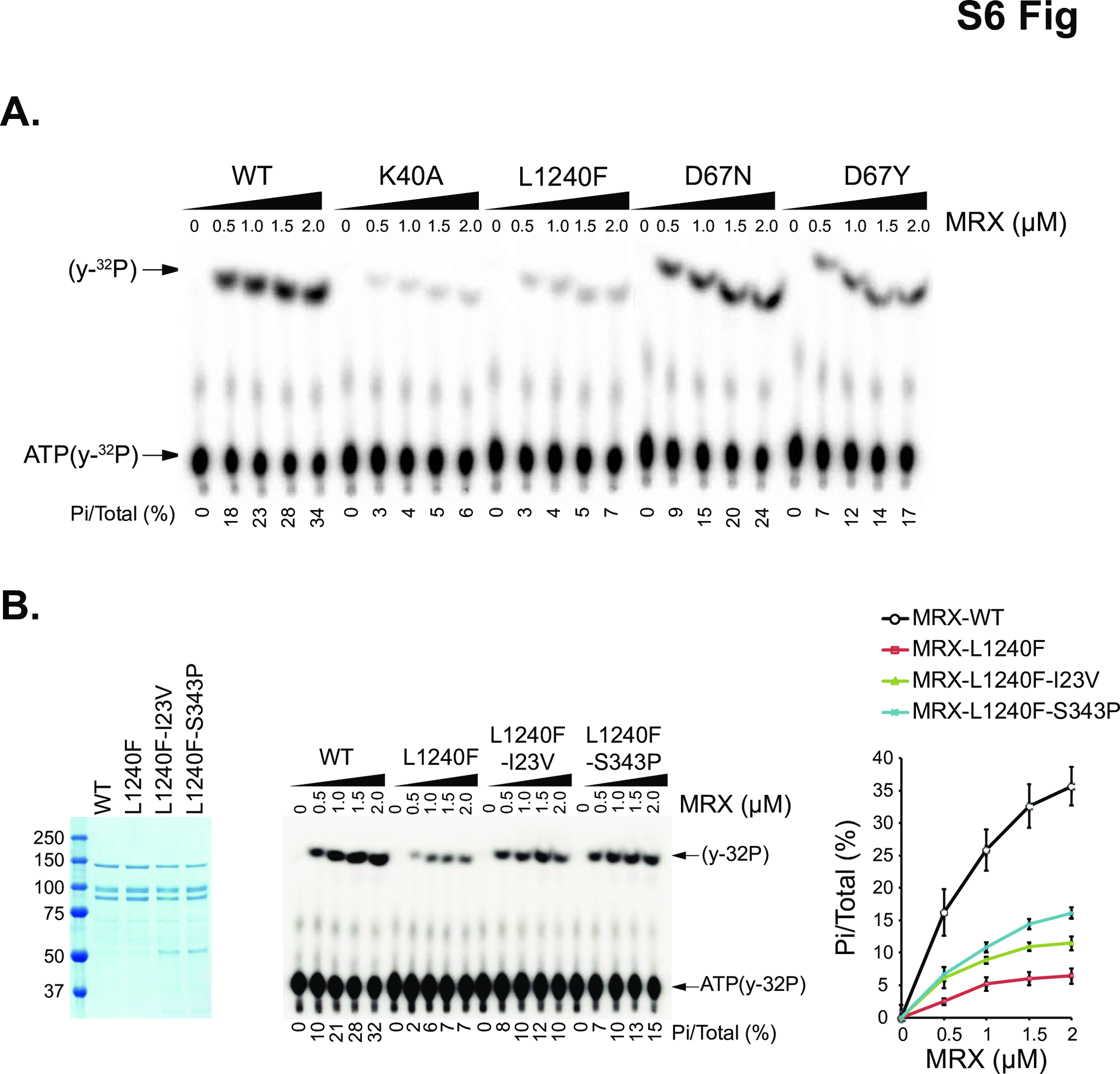

Supplement: S6 Fig — Figure related to Fig 4E. (A) ATPase activity of modeled mutants. Increasing concentrations of MRX complexes (0–2 μM) were incubated with γ32P-ATP- in presence of ssDNA and samples were run on a TLC plate. The migration levels of the hydrolyzed free phosphate (γ32P) and the non-hydrolyzed ATP (ATP-γ32P) are indicated. The signal intensity of γ32P and total signal per lane was quantified and the percent ATP hydrolysis (Pi/total) is given (bottom of TLC plate). Four independent experiments were quantified and are illustrated in graph shown in Fig 4E. (B) ATPase activity of Rad50-L1240F without and with I23V and S343P suppressors. 2 μg of purified Mre11 complexes were loaded on SDS-PAGE stained with Coomassie Blue. An example of an ATPase assay is shown. Standard deviations represent three experiments. (TIF) [file pgen.1008422.s006.tif]

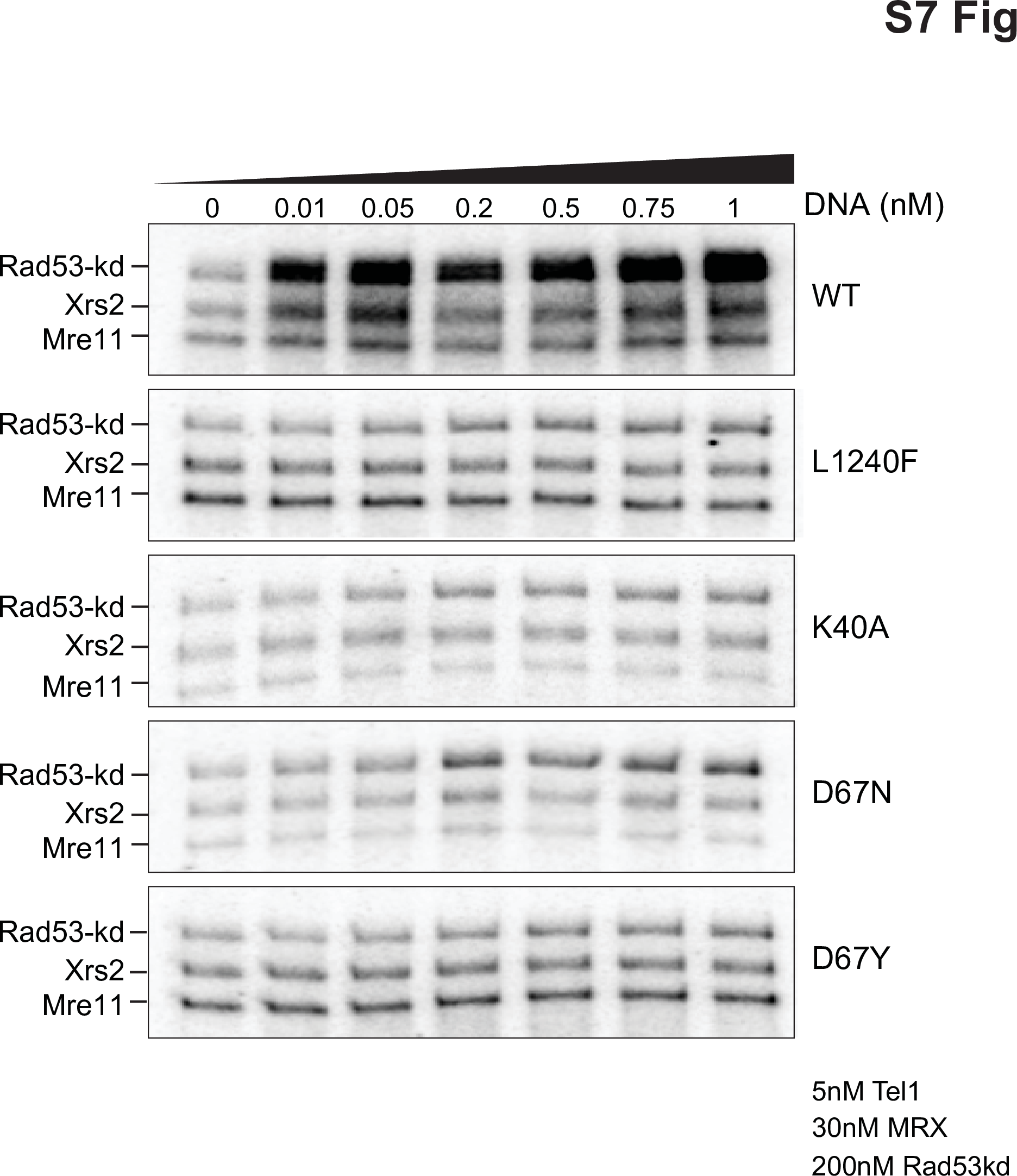

Supplement: S7 Fig — Figure related to Fig 4F. Standard kinase reactions contained 200 nM Rad53-kd and 50 μM [γ32P]-ATP in kinase buffer with or without 30 nM Mre11 complex and the indicated concentration of 2 kb linear DNA. Kinase reactions were initiated with 5 nM Tel1. Reactions were stopped after 15 min at 30 ˚C and analyzed by 7% SDS-PAGE, followed by phosphorimaging. (TIF) [file pgen.1008422.s007.tif]

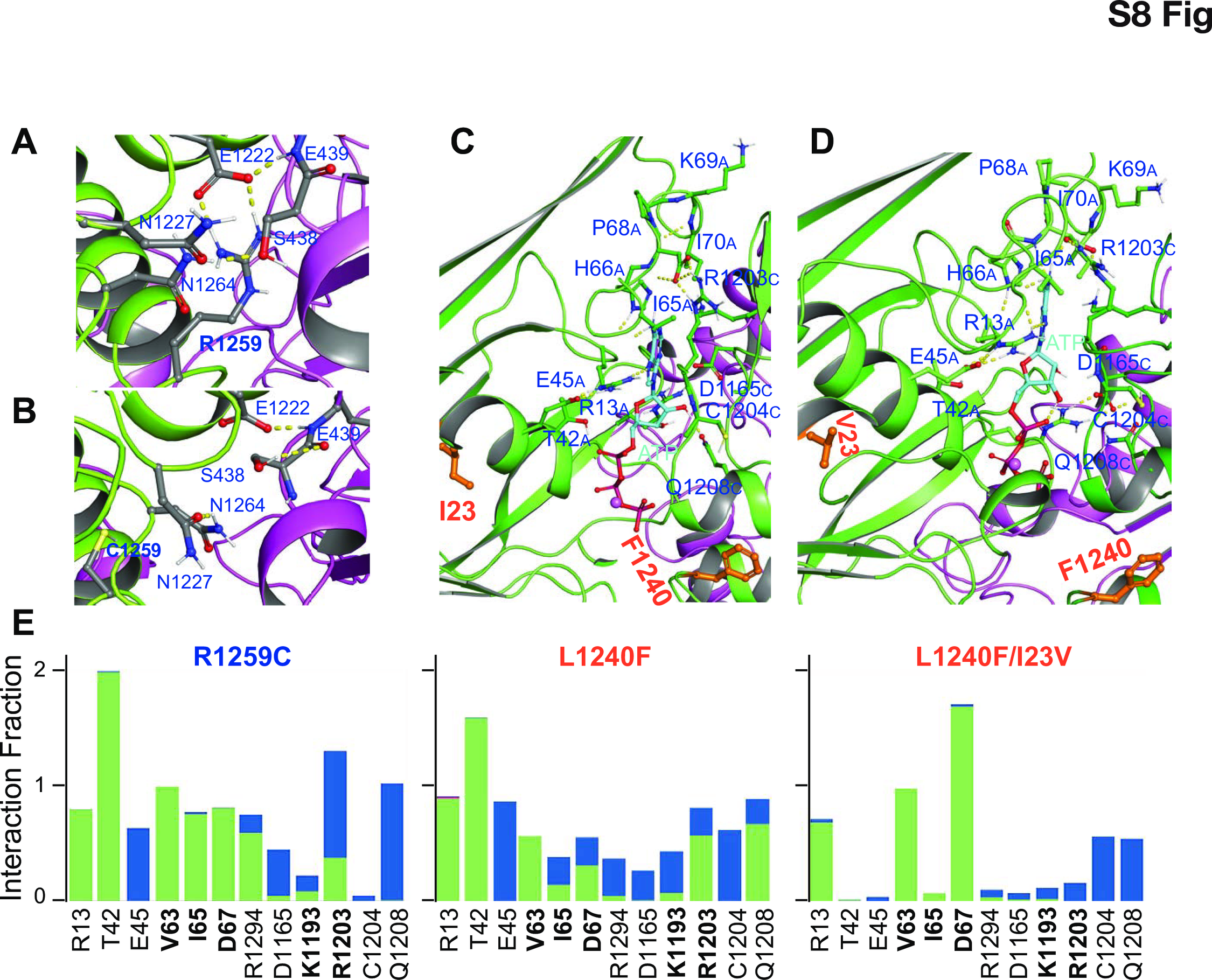

Supplement: S8 Fig — (A) Rad50-R1259-mediated interactions between the RBD domain of Mre11 (magenta) and Rad50 (green) molecules. (B) Impact of Rad50-R1259C mutant on interactions between the RBD domain of Mre11 (magenta) and Rad50 (green) molecules. (C) Rad50-L1240F mutation is localized far from the residues directly interacting with the adenine base, but closer to the triphosphate binding site. (D) The overall Mre11-Rad50 dynamics indirectly mediates the rescue effect of I23V on Rad50-L1240F mutant function. (E) Residue-specific protein-ATP interactions over the entire course of MD simulation in the vicinity of the Rad50-D67 residue. Amino acid residues that interact with the adenine base are highlighted in bold. (TIF) [file pgen.1008422.s008.tif]

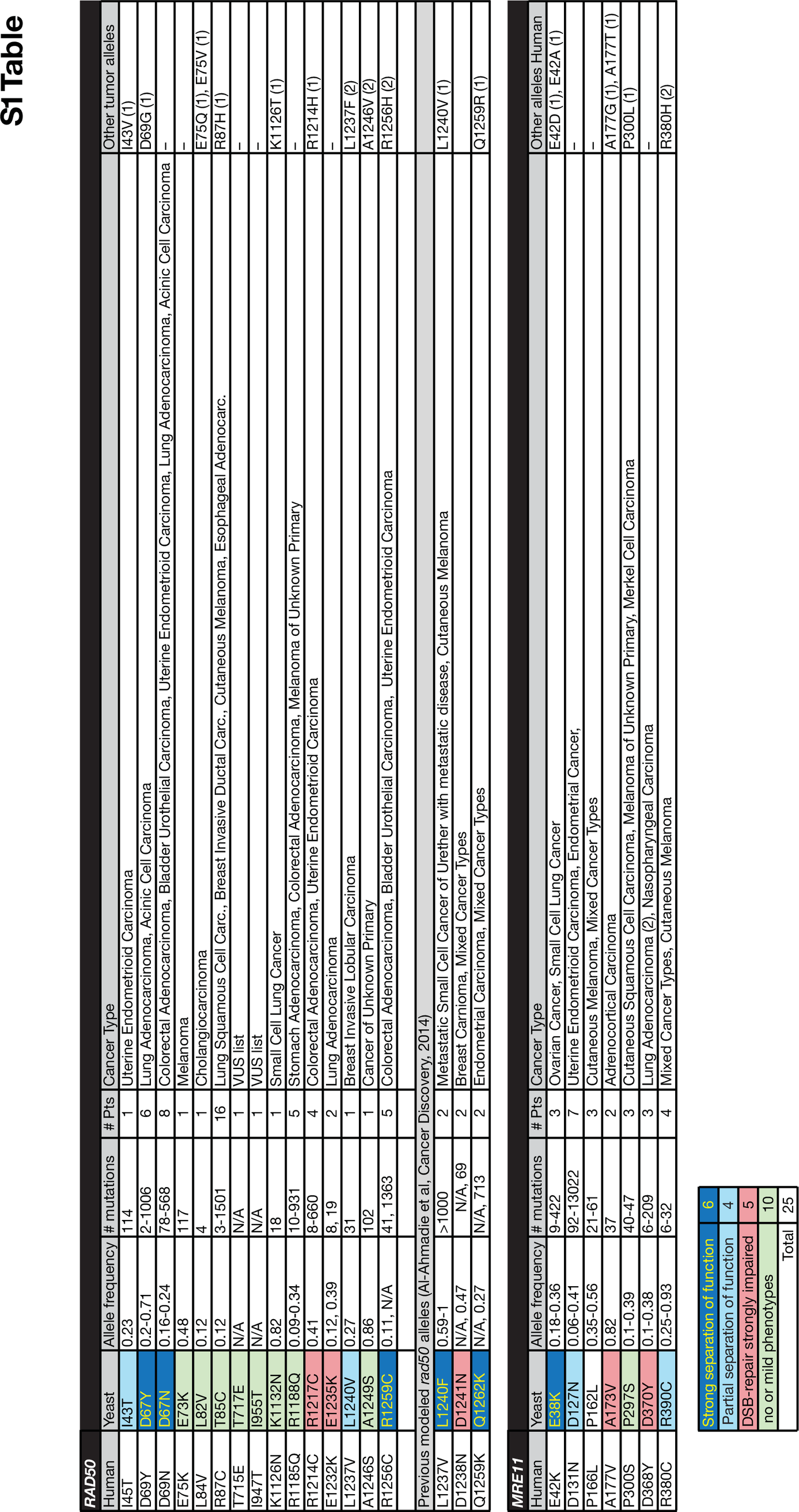

Supplement: S1 Table — The table list all alleles modeled in yeast. Separation of function (SOF) alleles (described in main text) are highlighted on dark blue (strong SOF alleles) or light blue (partial SOF alleles) background. Alleles deficient in DSB-repair (rad50Δ and mre11Δ alike, severe MMS-sensitivity in MEC1 background) are given on a red background. Alleles with only mild or no MMS-sensitivity are highlighted on a green background. Allele frequencies, number of mutations and tumor types are given. The alleles tested in this study are listed on the left side of the table. Some residues were also mutated in tumors to other amino acid residues (alleles given on right side of the table), but were not assessed in this study. (TIF) [file pgen.1008422.s009.tif]

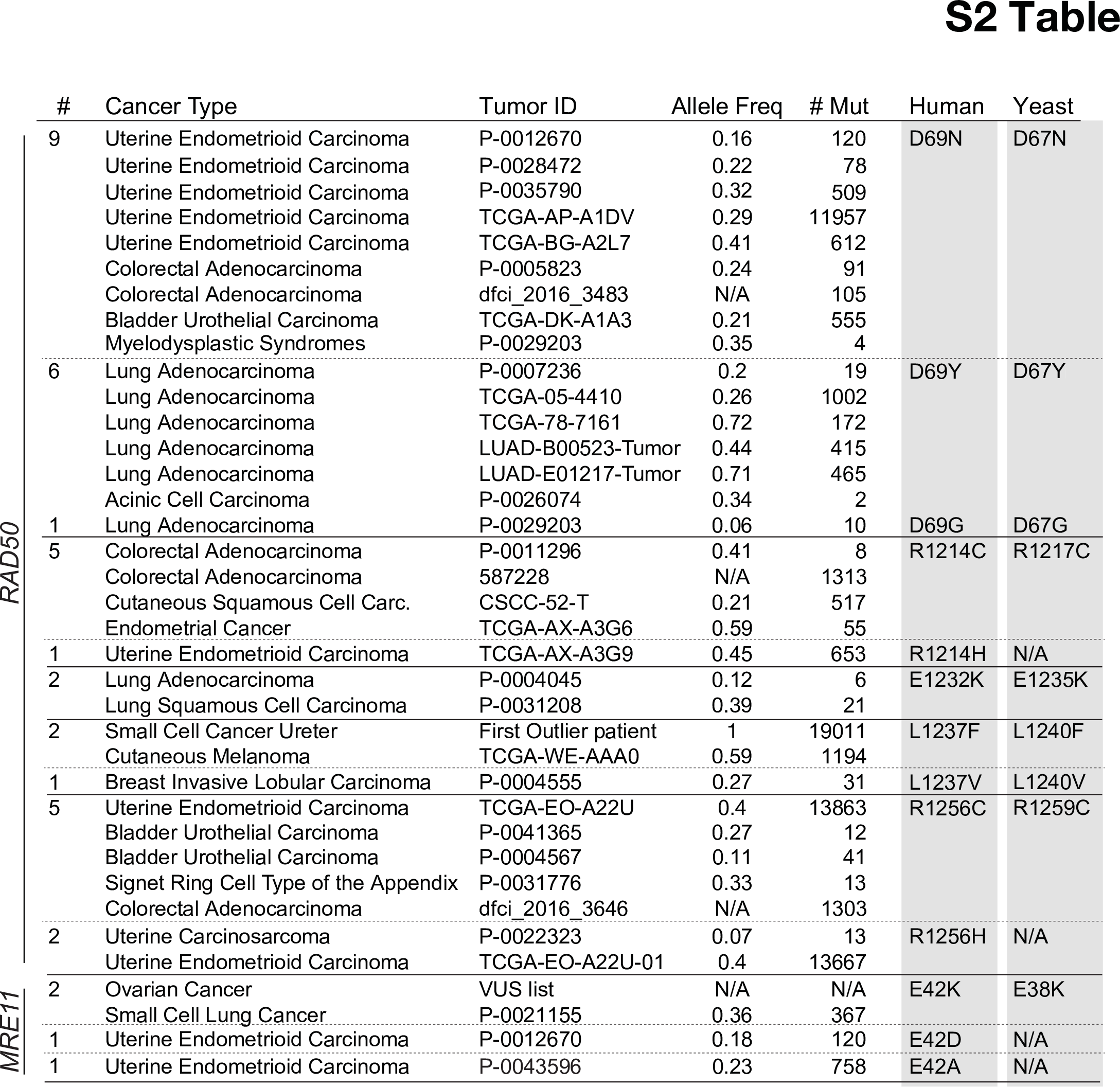

Supplement: S2 Table — Related to Fig 1A. This is an extended version of above the table shown in Fig 1A, listing all allele frequencies, number of mutations present in tumor samples and sequencing database source. (TIF) [file pgen.1008422.s010.tif]

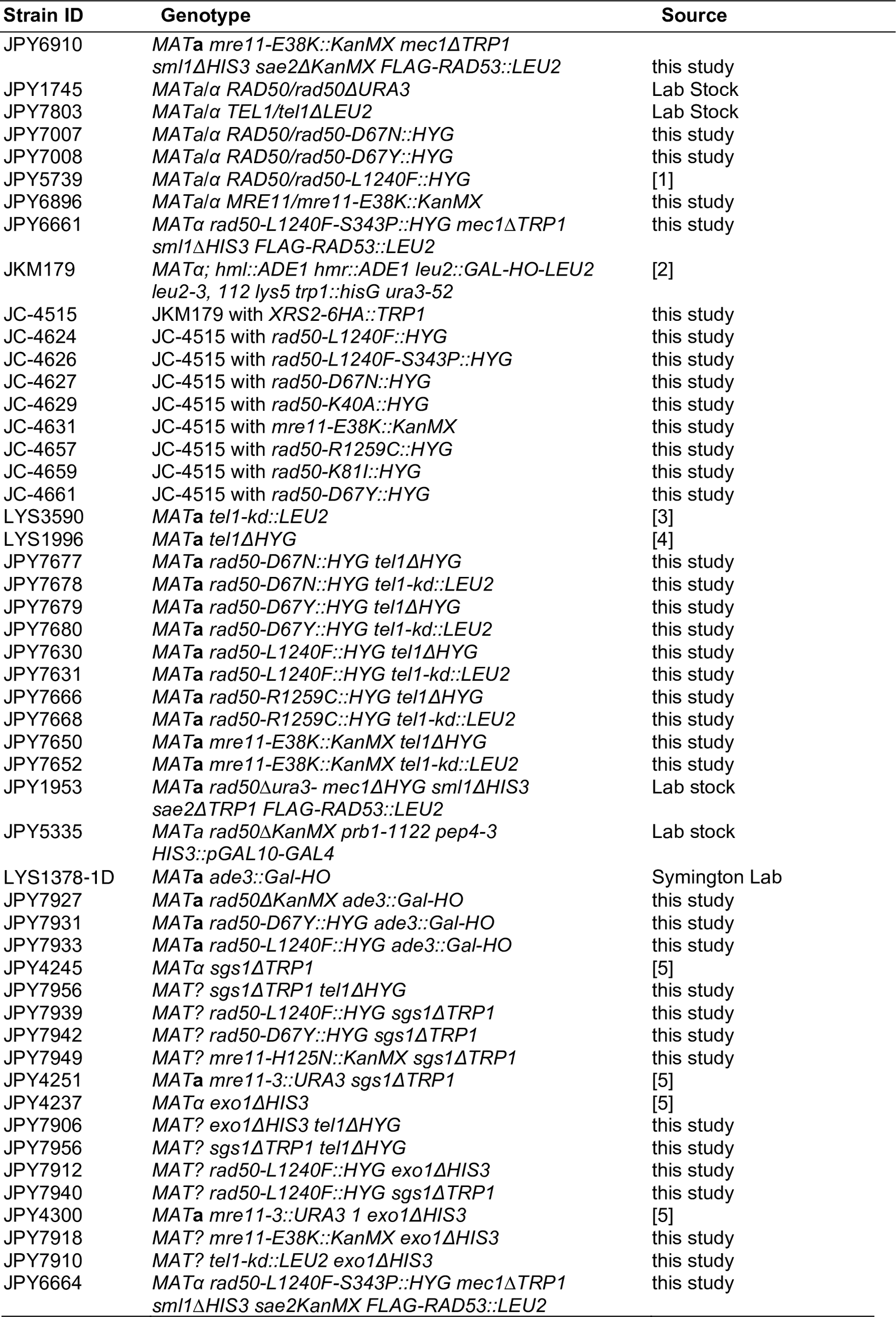

Supplement: S3 Table — (TIF) [file pgen.1008422.s011.tif]
