# Supplementary material for: Modeling cancer genomic data in yeast reveals selection against ATM function during tumorigenesis
Source: PLoS Genet. 2020 Mar 18;16(3):e1008422. doi: 10.1371/journal.pgen.1008422 (PMC7105138; doi:10.1371/journal.pgen.1008422)
Supplement: S1 Text — (DOCX) [file pgen.1008422.s017.docx]

**S1 Text**

**Supporting Methods**

**Gene conversion assay**

Mating type switching was assessed as previously described [65].

***rad50-L1240F* suppressor screen**

The *Ycp50-rad50-L1240F* plasmid was randomly mutagenized by propagating the plasmid in XL1 red bacterial mutator (*mutS, mutD, mutT*) strain (Agilent Technologies). *rad50Δ mec1Δ sml1Δ sae2Δ* spore (JPY1953) was transformed with the mutagenized *Ycp50-L1240F** plasmid library. 1000 larger *URA3* colonies were manually picked, diluted in water and assessed for growth on Do-Ura plates in presence of 2.5 μM CPT or 0.01% MMS. Eighteen colonies were able to grow in presence of either clastogen. Plasmids were recovered and retransformed in JPY1953, and the four plasmids promoting strongest suppression were sequenced. The identified suppressor mutants were by site directed mutagenesis de novo inserted in *rad50-L1240F::HYG* integration construct and integrated at the *RAD50* locus.
