# Supplementary material for: Modeling cancer genomic data in yeast reveals selection against ATM function during tumorigenesis
Source: PLoS Genet. 2020 Mar 18;16(3):e1008422. doi: 10.1371/journal.pgen.1008422 (PMC7105138; doi:10.1371/journal.pgen.1008422)
Supplement: S2 Text — Supporting text for S3 Fig. (DOCX) [file pgen.1008422.s018.docx]

**S2 Text**

**Supporting text for Fig S3: Intragenic Suppressors of *rad50-L1240F***

We previously showed that phenotypes of mutations affecting the Rad50 hook domain can be suppressed by intragenic mutations in the Rad50 coiled coil domain. This outcome is likely due to the intragenic mutations mitigating alterations in the path of the coiled coils caused by the hook domain mutations [8]. To gain insight regarding the mechanism(s) by which Tel1 activation is impaired in the *rad50* separation of function alleles, we carried out a screen for intragenic suppressors of *rad50-L1240F*. We mutagenized the *rad50-L1240F* ORF to establish a *rad50-L1240F** plasmid library. This library was transformed into a *rad50Δ mec1Δ sae2Δ* strain and the ensuing transformants were selected for MMS resistance. Four intragenic suppressors of *rad50-L1240F*, two in the coiled coil domain and one each in Walker A and B, were identified (*S343P*, *A1079T*, and *I23V*, *S1247N* respectively*;* see S3B Fig).

*rad50-L1240F-I23V, rad50-L1240F-S343P, rad50-L1240F-A1079T and rad50-L1240F-S1247N* strains were established in *mec1Δ* and *mec1Δ sae2Δ* backgrounds for assessment of MMS or CPT survival (S3A Fig) as well as Rad53 phosphorylation (S3C Fig). In each *rad50-L1240F* suppressor strain, survival on MMS and CPT were substantially enhanced (S3A Fig). This enhanced survival was associated with minimally increased Rad53 phosphorylation in *mec1∆* and *mec1∆* *sae2∆* strains (S3C Fig), suggesting that the intragenic suppressors partially restored the Tel1 activation defect of *rad50-L1240F*. Additionally, the mild telomere shortening phenotype of *rad50-L1240F* was partially suppressed by *rad50-S343P* and *rad50-A1079T,* but not by *rad50-I23V* and *rad50-S1247N* (S3D Fig). All four *rad50-L1240* suppressors also rescued the modest MMS and CPT sensitivity of *rad50-L1240F* observed at high clastogen doses in a Mec1-proficient setting (S3E Fig). Additionally, both *rad50-S343P* and *rad50-I23V* assessed, also fully rescued the mild meiotic phenotype of *rad50-L1240F* (S3F Fig), demonstrating that the suppressors both alleviate the Tel1 activation defect and the mild DNA repair phenotype of *rad50-L1240F.* Rad50 protein levels are partially reduced in *rad50-L1240F,* however both *rad50-S343P* and *rad50-I23V* suppressor did not effect Rad50 protein levels or interaction with Mre11 *(*S3G Fig).

*In vitro*, MRX-L1240F-S343P modestly increased ATP hydrolysis (S6B Fig). The Rad50 ATPase activity is required for enabling DNA access of the Mre11 active site to promote nuclease activity for DNA resection [2,38,39]. Consistent with the observed partial rescue of its ATPase activity, we found that *in vivo*, *rad50-L1240F-S343P* also rescued Mre11 complex DNA resection when assessed by Q-PCR at 0.15 kb and 4.8 kb from the HO-DSB (S3H Fig).

These data indicate that, as with *rad50^hook^* mutations [8], a mutation in the globular domain (*rad50-L1240F*) can be suppressed by mutations in the coiled coil domain, as well as also by *rad50-L1240F* proximal and distal mutations within the globular domain.
