# Supplementary material for: Modeling cancer genomic data in yeast reveals selection against ATM function during tumorigenesis
Source: PLoS Genet. 2020 Mar 18;16(3):e1008422. doi: 10.1371/journal.pgen.1008422 (PMC7105138; doi:10.1371/journal.pgen.1008422)
Supplement: Attachment — Submitted filename: Petrini_Response to Reviewers.docx [file pgen.1008422.s019.docx]

**Response to Reviewers:**

**In General**: We are gratified that the Reviewers appreciate the quality (and quantity) of the data that we present to address the role of the Mre11 complex in tumor suppression. All the reviewers agreed that the work has the potential to provide important insights. Moreover, it is clear from their detailed and insightful comments that they did a careful and thorough job with the reviews for which we are grateful. As a result of their input, we’ve added several pieces of data, clarified the underlying logic of our interpretations, and the paper is much improved as a result. Hopefully, you will agree that is now suitable for the readership of PLOS Genetics.

It is clear that we failed to adequately convey certain key concepts that underlie our interpretations and as a result, the reviewers raised appropriate concerns. I will address these here, and will subsequently address the specific concerns of each Reviewer individually. In response to the Reviewers’ concerns, the following data were added to the main Figures and to the Supplemental information: **[Fig 2F, Fig 4B, S1A Fig, S1B Fig, S3C Fig, S3H Fig, S4 Fig]**. In the paragraphs below, we refer to the figures listed here. In addition, we include one Figure (Figure R1) solely for the reviewers. We don’t feel that it warrants inclusion in the manuscript, but we hope it helps clarify the SOF argument. This is described in point #5 below. Finally, literature cited in this response is listed at the end of document.

**First**, Reviewers 1 and 2 both noted that Tel1 deficiency dramatically sensitized *rad50-L1240F* and *mre11-E38K* to CPT and suggested that this lack of epistasis belies our interpretation of separation of function.

Respectfully, we suggest reexamining that interpretation. Epistasis between hypomorphic and null alleles of a given pair of genes must be interpreted differently than that between a pair of null mutants.

In the former circumstance (such as in this paper), the phenotypic severity of the double mutant may be greater than that of either single if the genes in questions function in the SAME pathway. For example, this would be true if the gene products of the two genes in question physically interact, and if the absence of one gene product renders the mutant gene product inactive.

In the latter situation, the logic of which the Reviewers seem to be applying in their assessment of this paper, two *null* mutants that function in the same pathway give the same phenotype as either of the individual mutants. Applying that logic would indeed predict that the SOF alleles should phenocopy Tel1 deficiency.

*rad50-L1240F* and *mre11-E38K* are not null mutations and we propose that their interaction with Tel1 protein is critical to their function. This is an example of the first circumstance described above.

The Rad50-L1240F and Mre11-E38K-containing complexes depend on the presence of Tel1 protein for DNA repair functions. We propose that the difference in *tel1∆* strains vs. *tel1KD* reflects that the *rad50-L1240F* and *mre11-E38K* gene products interact with Tel1 protein and are stabilized by that interaction.

As Reviewer #1 states, the difference between *tel1∆* and *tel1KD* indicates substantial Tel1 function in *rad50-L1240F*. Formally speaking, this is true, *but we propose that the relevant function in this context is binding to Rad50.* We state this interpretation more forcefully in the revised version.

Supporting this concept:

1. *rad50-L1240F* and *mre11-E38K* exhibit other phenotypes that support the view that their respective complexes are unstable.
   a. They exhibit markedly enhanced sensitivity to CPT at 37°. This is not true of *rad50-D67N* or *rad50-D67Y*, two SOF alleles that exhibit the same clastogen sensitivities in *tel1∆* and *tel1KD* (*i.e.*, they do NOT depend on Tel1 protein for DNA repair functions). **[S4 Fig]**
   b. As noted in the original submission, Rad50-L1240F-containing complexes are the poorest binders of ATP and DNA *in vitro*. As ATP binding contributes to the structure of the complex, this likely contributes to the inherent instability.
   c. *tel1KD* *rad50-L1240F* or *tel1KD* *mre11-E38K* double mutants are as CPT or MMS resistant as either single *tel1KD* or *rad50-L1240F*, *mre11-E38K* and *xrs2-664* (the Xrs2 C terminal truncation [1] mutant invoked by Reviewer #2).

Supporting the interpretation of separation of function:

2. Like *tel1∆* and *tel1KD* single mutants, *rad50-L1240F* and *mre11-E38K* are mildly sensitive only to extremely high doses of CPT (20 and 40µM). For comparison, the *mre11-H125N* mutant, which is proficient in mating type switching [2] exhibits considerably higher CPT and MMS sensitivity than the SOF alleles presented here. We note this in the text and this point is emphasized in the revised version. We consider these two alleles to be SOF since they are significantly more defective in Tel1 activation by several criteria than they are in DSB repair. We highlight the modest defects observed in the text when discussing clastogen sensitivity and NHEJ and reemphasize that high doses of clastogen are required to see sensitivity in these mutants.
3. Rad50-L1240F and Rad50-D67N/Rad50-D67Y containing complexes are completely dead for Tel1 activation *in vitro* and exhibit very limited activation *in vivo*.
4. DNA resection is only very modestly impaired in the SOF mutants. First, *rad50-L1240F* *exo1∆* double mutants exhibit the same CPT and MMS sensitivities as *rad50-L1240F* *tel1KD*. Second, whereas Mre11 nuclease dead mutants markedly enhance the CPT sensitivity of *sgs1∆* [3], the *rad50-L1240F* *sgs1∆* exhibits similar sensitivity as *sgs1∆* *tel1KD* double mutants **[S1B Fig]**. To examine resection physically, we carried out Q-PCR analyses. By this approach, we do see a modest reduction in the *rate* of resection **[Fig 2F],** but this kinetic difference does not have a major impact on survival after clastogen exposure. It is worth noting previous data from Yunji Cho’s lab suggesting that ATP hydrolysis by Rad50 drives a structural transition that opens the globular domain and renders the Mre11 nuclease active site accessible [4]. And, the magnitude of the resection defect in SOF mutants revealed by Q-PCR scales with the magnitude of ATP binding and hydrolysis defects observed *in vitro*. This likely explains the kinetic differences observed.
5. The *tel1-HY* allele [5] exhibits chronic Tel1 activation. When combined with *WT* *RAD50* and the SOF mutants, the MMS sensitivity of all the ensuing double mutants is essentially identical, save for the slight sensitivities of *rad50-L1240F* and *mre11-E38K* observed at high MMS doses previously. This would not be true if significant DSB repair defects were manifest **[Fig R1]**.

**Reviewer #1**. Reviewer #1’s concerns are addressed above.

**Reviewer #2**.

1. In two instances, this Reviewer states that ATM is required for resection and ATR activation. Several lines of evidence argue against this view. In neither human cells nor budding yeast does ATM deficiency cause strong sensitivity to HU. *tel1∆* cells have only a very subtle defect in end resection [6], and DSB-induced Rad53 phosphorylation is essentially wild type in *tel1∆* [6]. Cells and tissues from ATM deficient mice do not exhibit HR defects [7], and ATM deficient cells do not exhibit any defects in ATR dependent RPA or Chk1 phosphorylation [8]. Steve Jackson published a paper asserting that ATM was required for ATR activation in 2006 [9]. Nevertheless, the evidence against that conclusion accrued over the subsequent 13 years is substantial. Although that context argues strongly against a role for Tel1/ATM in Mec1/ATR activation, we tested HU sensitivity of *rad50-L1240F*, *rad50-D67N* and *mre11-E38K*. As expected, these strains do not exhibit sensitivity at the highest dose tested (200mM HU) **[Fig 2A]**.
2. This reviewer suggests that we examine resection and HR events physically as we have done for NHEJ. The resection issue has been addressed above. WRT HR, since NHEJ deficiency has virtually no effect clastogen sensitivity, survival upon challenge with clastogens inherently reflects HR proficiency. Physical assays employing an HO break offer a less sensitive readout. For example, as noted above, the *mre11-H125N* mutant is much more sensitive to CPT than our SOF mutants, but scores as *WT* in a mating type switching assay [2]. Similarly, the *mre11-D56N* mutant (also nuclease dead) is not defective in mating type switching, but is sensitive to IR or to the induction of multiple HO breaks [10]. For these reasons, we feel that the mating type assay is not a sensitive assay to measure subtle differences in HR. Nevertheless, we carried out a mating type switching assay and as expected, the behavior of *rad50-L1240F* and *rad50-D67Y* was indistinguishable from *WT* **[S1A Fig]**.
3. We added a comment in the results section to clarify the apparent discrepancy between DNA binding *in vitro* by Rad50 and the ChIP data (comment #8).

**Reviewer #3.**

This reviewer offered several suggestions for improving the flow of the manuscript which we incorporated. The suppressor data was moved to the supplemental information as suggested **[S3 Fig]**. Language usage issues (*i.e.*, lack of a clear antecedent in some cases) were addressed. And, a careful edit was carried out for clarity and concision. We are grateful to this Reviewer for thoughtful treatment of our manuscript.

**Literature cited**

1.	Shima, H., M. Suzuki, and M. Shinohara, *Isolation and characterization of novel xrs2 mutations in Saccharomyces cerevisiae.* Genetics, 2005. **170**(1): p. 71-85.

2.	Moreau, S., J.R. Ferguson, and L.S. Symington, *The nuclease activity of Mre11 is required for meiosis but not for mating type switching, end joining, or telomere maintenance.* Mol Cell Biol, 1999. **19**(1): p. 556-66.

3.	Foster, S.S., A. Balestrini, and J.H. Petrini, *Functional interplay of the Mre11 nuclease and Ku in the response to replication-associated DNA damage.* Molecular and Cellular Biology, 2011. **31**(21): p. 4379-89.

4.	Liu, Y., et al., *ATP-dependent DNA binding, unwinding, and resection by the Mre11/Rad50 complex.* EMBO J, 2016. **35**(7): p. 743-58.

5.	Baldo, V., et al., *Dominant TEL1-hy mutations compensate for Mec1 lack of functions in the DNA damage response.* Mol Cell Biol, 2008. **28**(1): p. 358-75.

6.	Mantiero, D., et al., *Dual role for Saccharomyces cerevisiae Tel1 in the checkpoint response to double-strand breaks.* EMBO Rep, 2007. **8**(4): p. 380-7.

7.	Kass, E.M., et al., *Double-strand break repair by homologous recombination in primary mouse somatic cells requires BRCA1 but not the ATM kinase.* Proc Natl Acad Sci U S A, 2013. **110**(14): p. 5564-9.

8.	Balestrini, A., et al., *Defining ATM-Independent Functions of the Mre11 Complex with a Novel Mouse Model.* Mol Cancer Res, 2016. **14**(2): p. 185-95.

9.	Jazayeri, A., et al., *ATM- and cell cycle-dependent regulation of ATR in response to DNA double-strand breaks.* Nat Cell Biol, 2006. **8**(1): p. 37-45.

10.	Llorente, B. and L.S. Symington, *The Mre11 nuclease is not required for 5' to 3' resection at multiple HO-induced double-strand breaks.* Mol Cell Biol, 2004. **24**(21): p. 9682-94.
