# Supplementary material for: Modeling cancer genomic data in yeast reveals selection against ATM function during tumorigenesis
Source: PLoS Genet. 2020 Mar 18;16(3):e1008422. doi: 10.1371/journal.pgen.1008422 (PMC7105138; doi:10.1371/journal.pgen.1008422)
Supplement: Attachment — Submitted filename: Hohl et al 2nd rebuttal.doc [file pgen.1008422.s020.doc]

Dear Dr. Symington

Thank you for your careful consideration of our manuscript. We are once again grateful for the careful attention of the Reviewers to the paper.

Comments to Dr. Symington (in blue).

Reviewer 1 is still concerned that the separation of function phenotype is overstated because the *rad50-L1240F* and *mre11-E38K* mutants are not WT for DNA repair functions (we revised the text and stated that these mutants have a partial defect in DNA repair), and *mre11-E38K* synergizes with *tel1-kd*, in contrast to what is stated in the text (that was a clear mistake and we corrected that). Sporulation and spore viability of the *rad50-L1240F* mutant seem quite a bit lower than WT. Please can you add p values to Figs 2D. (we added P-values to Fig 2D). I find it quite surprising that telomere length is not more strongly affected by the mutations, suggesting that they are not completely compromised for Tel1 activation. We were surprised about that first too. To our knowledge, currently we do not know how much Tel1 activity is required for telomere length maintenance. We speculated that lower levels of Tel1 activity may be needed to maintain normal telomere lengths than for Mre11 complex function in Tel1 signaling and accordingly one sentence in the result section (Page 11).

We also noted that Rad53-phosphorylation/survival in *mec1∆* background and telomere lengths do not always correlate, suggesting that telomere length maintenance is also aside from Tel1 activity also dependent on other unknown molecular functions.

Ma and Greider (2009, **DOI:** 10.1128/MCB.01896-08) have shown that Xrs2-dependent recruitment of the Tel1 protein has some kinase independent functions in telomere length maintenance. Thus, possibly the increased Tel1-recruitment observed for *rad50-L1240F,* or an altered MRX-Tel1 interaction itself*,* could have some impact in this regard as well.

I think part of the problem with making general conclusions about the mutations is that each behaves a little differently in the various assays described, reflecting the complex functions of the MRX/N in DNA repair and signaling. This is an issue inherent to all genetic analyses. It is especially difficult when addressing the functions of a functionally diverse protein complex. Nevertheless, we feel that our work on these mutants provides novel and useful insights and that this paper is a contribution that will edify the broad readership of PLOS Genetics. We are very hopeful that you agree.

With sincere thanks for your careful stewardship of this paper,

Jp

John H. J. Petrini Ph. D.

Member and Chair, Molecular Biology Program

Paul A. Marks Chair of Molecular Cell Biology

Memorial Sloan Kettering Cancer Center

1275 York Ave

RRL 901C

New York, NY 10065

212 639 2927 (office)

646 422 2062 (FAX)

[petrinij@mskcc.org](mailto:petrinij@mskcc.org)

<http://ski.edu/petrini>

1) Provide a detailed list of your responses to the review comments and a description of the changes you have made in the manuscript.

- The changes described above were made on pages 7, 11, 12, 22.
- We fixed an error in Fig 4B (noted below in the response to Reviewer 1).
- Fixed the typos of unclear antecedents noted by Reviewer #3.
- Added a sentence to the discussion as suggested by Reviewer #3.
- Made several minor revisions of the text throughout the Results and Discussion to increase clarity and to revise overstatements.

**Reviewer's Responses to Questions** (in blue)

Reviewer #1: My previous primary criticism, which was shared with another reviewer, was that key rad50/mre11 alleles reported in this paper did not behave as straightforward SOF mutations that ablate MRX-dependent Tel1 function without impairing MRX DNA repair functions.

We agree with this reviewer, that *rad50-L1240F* and *mre11-E38K* partially affected the DNA repair functions (most pronounced in CPT-survival and in meiosis) and thus the separations of DNA repair and Tel1/ATM activation is less clean in these mutants.

Thus we added the following sentence in Page 7:

“The separations of DNA repair and Tel1/ATM activation in that class of mutant was less pronounced than in the second class; both *mre11-E38K* and *rad50-L1240F* exhibited partial defects in DNA repair”.

I noted that their effects in DNA repair assays (e.g. CPT sensitivity) were strongly additive with a *tel1* deletion mutation. The authors reply that epistasis tests may be misinformative with these alleles because they are not simple null mutations. They propose that the alleles in question destabilize the MRX complex to make its DNA repair function dependent on a physical interaction with Tel1. The principal reason for proposing this model is that they believe the alleles show little or no synergism with a *tel1KD* (kinase dead) alleles. In point C of “supporting this concept”, the rebuttal states: “*tel1KD rad50-L1240F* or *tel1KD mre11-E38K* double mutants are as CPT or MMS resistant as either single *tel1KD* or *rad50-L1240F*, *mre11-E38K* and *xrs2-664* (the Xrs2 C terminal truncation [1] mutant invoked by Reviewer #2).”

We apologize for this obvious mistake in our statement. *mre11-E38K* shows clearly strong synergism with *tel1-kd*, to comparable levels as *tel1∆*. Thanks for spotting that.

However, to my eyes the data in Figure 4 contradict this statement. From the 5-fold serial dilutions on 25 micromolar CPT, tel1KD rad50-L1240F appears to be about 5-fold more sensitive than tel1KD and >25-fold more sensitive than rad50-L1240F.

We completely agree. A 5-fold difference in survival of *rad50-L1240F tel1-kd* in comparison to *tel1-kd* is relatively mild at this high dose of 25 µM CPT.

*rad50-L1240F tel1∆* in stark contrast shows at 10 µM CPT about 125-fold less survival than *rad50-L1240F tel1-kd* and thus clearly demonstrates that *rad50-L1240F* CPT-survival depends much more on the presence of the Tel1 protein rather than its kinase activity.

Finally, it bears some consideration that these are very high doses of clastogen. For example, a *rad50∆* is completely dead at 5 µM CPT. We used 25 µM CPT to stringently assess DNA repair capacity.

Similar differences are obvious for 0.020 micromolar MMS (it should have been labeled 0.020% MMS; that was a mislabeling in the Fig 4B; we corrected that mistake, thank you) and 200 mM HU.

Likewise, tel1KD mre11-E38K is clearly much more sensitive to CPT, MMS and HU than either tel1KD or mre11-E38K single mutants (Figure 4).

We agree with that and corrected it.

DSB repair of *rad50-L1240F* and *mre11-E38K* is dependent on the Tel1 interaction. We added a sentence in Page 13:

“Therefore, these data indicate that *rad50-L1240F* and *mre11-E38K* depend on interaction with Tel1 protein for DSB repair, and their defect in Tel1 activation is attributable to an intrinsic molecular function”.

(Side note: I cannot find any data for xrs2-664 in the manuscript, so it is unclear how the authors know how it compares to tel1KD rad50-L1240F or tel1KD mre11-E38K).

We only mentioned this *xrs2* mutant to respond to the reviewers comment. We did not use that strain in our study. We just approximately compared the survival of *rad50-L1240F tel1-kd* at 20 µM CPT with the survival at same CPT dose of *xrs2-664* published by Shinohara’s lab (Shima et al (2005):Genetics 170: 71-85) - Fig 3C shows that *xrs2-664* is slightly more CPT-sensitive than *WT* strain, similar as *tel1∆* or *tel1-kd* in in our hands.

The rebuttal further states: “We consider these two alleles to be SOF since they are significantly more defective in Tel1 activation by several criteria than they are in DSB repair”. I would not feel confident in drawing this conclusion from these data. There is no objective or unambiguous way to compare the assays.

If I were to propose that the rad50-L1240F or mre11-E38K mutations have reduced MRX-dependent Tel1 activation and DNA repair activities both by about 75%, I cannot identify any data in this manuscript that would convincingly disprove this hypothesis.

It’s hard to see how a 75% decrease in Mre11 complex DNA repair activities is consistent with the data in this paper. At the doses of MMS and CPT we use here, a *rad50∆* strain shows no growth at any dilution (e.g., Hohl et al, 2015; Mol Cell 57, pp1-13).

That said, we agree that there is indeed no unambiguous way to compare the assays. We can only compare the phenotypic outcomes to other mutants in Mec1-proficent and Mec1-deficient background.

Both *rad50-L1240F* and *mre11-E38K* partial DSB repair defect is much milder than for example *mre11-H125N* (the Mre11 nuclease dead allele). *mre11-H125N* in contrast is not required for Tel1 activation-both survival and Rad53-phosphorylation are similar to *MRE11 WT*. This is clearly not the case for *rad50-L1240F* and *mre11-E38K*, in which both in Mec1-deficent background Rad53 phosphorylation and clastogen survival are very low.

Also, MMS-survival of *rad50-L1240F mec1∆* and *mre11-E38K mec1∆* are almost completely rescued by a tel1-hy allele (we provided this data in first revision). This would not be true if significant DSB repair defects were manifest.

Reviewer #3: The ms. has been improved significantly and is much easier to read. Thanks for this acknowledgment.

There is still some "roughness" in the presentation of the molecular phenotype and biochemical data concerning the SOF mutations.

We did our best to improve the presentation. As noted above, we carried out “tweaks” throughout the manuscript to improve clarity, at this reviewer’s suggestion. The paper is still quite dense partially as a result of the amount of data. Hopefully we’ve made it still easier with the current revision.

Given the concerns of two of the reviewers, this section needs a little more attention to make it clear what can and cannot be said about the SOF phenotype of each allele.

We followed reviewer 1 suggestions and revised some of our statement about the SOF phenotypes that some of them also partially affected the DNA repair functions.

The real take-home message is that MRN mutations play a complex tumor suppressor role. Indeed it is quite clear that the recombination function is likely not important for this role. It might be worthwhile to emphasize this result in the title and indicate that DNA repair functions of the MRN complex are not necessary for tumor suppression.

We agree with this statement that DNA repair is likely not important for the Mre11 complex role in tumor suppression. We wish though to do first some follow up studies in mouse to be sure about this preliminary conclusion. A sentence was added in the discussion to emphasize this point.

Typos:

pg. 18, ln. …as well biochemical… -> …as well as biochemical*…=> corrected, thank you.*

pg. 19, ln. 12: …activation defects Rad50-1240F… -> …activation defects in (or of) Rad50-1240F => corrected, thank you.

pg. 14, ln. 11; pg. 19, last ln.; pg. 20, ln. 3; pg. 47 last ln.: In each case, "This 'what?'”

=> clarified in each case. Thank you.

pg. 21, 6th ln. from bottom: “)” has no partner “(“ => corrected, thank you*.*
